# Arterial function in response to a 50 km ultramarathon in recreational athletes

**DOI:** 10.1113/EP091680

**Published:** 2024-06-11

**Authors:** Sushant M. Ranadive, Cynthia M. Weiner, Lauren E. Eagan, Odessa Addison, Rian Q. Landers‐Ramos, Steven J. Prior

**Affiliations:** ^1^ Department of Kinesiology University of Maryland College Park Maryland USA; ^2^ Department of Physical Therapy and Rehabilitation Science University of Maryland Baltimore Maryland USA; ^3^ Department of Veterans Affairs and Veterans Affairs Medical Center, Geriatric Research Education and Clinical Center Baltimore Maryland USA; ^4^ Department of Kinesiology Towson University Towson Maryland USA

**Keywords:** endothelial function, flow‐mediated dilatation, inflammation, wave‐reflection

## Abstract

This study was performed to determine whether prolonged endurance running results in acute endothelial dysfunction and wave‐reflection, as endothelial dysfunction and arterial stiffness are cardiovascular risk factors. Vascular function (conduit artery/macrovascular and resistance artery/microvascular) was assessed in 11 experienced runners (8 males, 3 females) before, during and after a 50 km ultramarathon. Blood pressure (BP), heart rate (HR), wave reflection, augmentation index (AIx) and AIx corrected for HR (AIx75) were taken at all time points—Baseline (BL), following 10, 20, 30 and 40 km, 1 h post‐completion (1HP) and 24 h post‐completion (24HP). Flow‐mediated dilatation (FMD) and inflammatory biomarkers were examined at BL, 1HP and 24HP. Reactive hyperaemia area under the curve (AUC) and shear rate AUC to peak dilatation were lower (∼75%) at 1HP compared with BL (*P *< 0.001 for both) and reactive hyperaemia was higher at 24HP (∼27%) compared with BL (*P* = 0.018). Compared to BL, both mean central systolic BP and mean central diastolic BP were 7% and 10% higher, respectively, following 10 km and 6% and 9% higher, respectively, following 20 km, and then decreased by 5% and 8%, respectively, at 24HP (*P *< 0.05 for all). AIx (%) decreased following 20 km and following 40 km compared with BL (*P *< 0.05 for both) but increased following 40 km when corrected for HR (AIx75) compared with BL (*P* = 0.02). Forward wave amplitude significantly increased at 10 km (15%) compared with BL (*P* = 0.049), whereas backward wave reflection and reflected magnitude were similar at all time points. FMD and baseline diameter remained similar. These data indicate preservation of macrovascular (endothelial) function, but not microvascular function resulting from the 50 km ultramarathon.

## Abstract (highlights)

- **What is the central question of this study?**
  What are the acute effects of prolonged distance running (50 km race) on endothelial function, wave reflection and inflammation both during a distance race and within 24 h after completion of the race?
- **What is the main finding and its importance?**
  Even though macrovascular function remains unchanged after a 50 km ultramarathon, microvascular function is attenuated. However, the microvascular changes lasted less than 24 h post‐race suggesting the ultramarathon distance of 50 km has relatively short‐term effects in trained individuals.

## 1 INTRODUCTION

Individuals who participate in aerobic exercise have lower cardiovascular disease (CVD) risk compared with their sedentary counterparts (Mora et al., 2007; Richardson et al., 2004). The cardioprotective effects of habitual aerobic exercise can, in part, be attributed to reduced chronic systemic inflammation, improved arterial structure and improved endothelial function (Autenrieth et al., 2009; Ford, 2002; Green et al., 2012; Kullo et al., 2007). Paradoxically, acute aerobic exercise also provokes a systemic inflammatory response similar to one seen in an acute immune response with the magnitude change proportionate to the duration of the event and/or degree of muscle damage (Bird et al., 2014; Marklund et al., 2013; Pedersen et al., 1998). The exaggerated systemic inflammatory response is observed in ultra‐endurance bouts of exercise, which vary in length, but are generally defined as longer than a marathon (>26.2 miles; 42.2 km) (Bonsignore et al., 2017). In this context we have reported elevated inflammatory makers (interleukin‐6 (IL‐6) and calprotectin) immediately post‐race for the 50 km distance ultramarathon (Landers‐Ramos et al., 2022).

Systemic inflammation, even if acute, induces endothelial dysfunction by reducing nitric oxide (NO) bioavailability and augmenting oxidative stress (Clapp et al., 2004). Furthermore, systemic inflammation and augmented oxidative stress can independently and together affect arterial stiffness via chronic endothelial dysfunction and heightened sympathetic modulation. In this context, arterial stiffness is independently associated with increased risk of CVD (Vlachopoulos et al., 2005). Increases in large artery stiffness, but not small artery stiffness, have been observed after an ultramarathon (Burr et al., 2012). In addition, those who regularly participated in ultra‐exercise compared with recreationally active controls were shown to have heightened large artery stiffness at rest potentially suggesting a change in arterial structure (Burr et al., 2014). These potential changes in endothelial function and arterial structure may influence wave reflections, particularly when combined with arterial stiffening that can occur during ultramarathons/prolonged exercise. Therefore, it is suspected that prolonged endurance athletes, such as ultramarathon runners, could be at a higher risk for cardiovascular events due to the increase in artery stiffness both at rest and acutely after an ultramarathon (Burr et al., 2012; Burr et al., 2014). Recently, an elaborate study by King et al. (2021) evaluated endothelial function at the femoral artery and inflammatory cytokines before and following 25, 50, 100 and 200 km races. However, changes in arterial function and inflammatory cytokines during and 24 h following a 50 km ultramarathon and their relationships to endothelial function at the brachial artery in recreational athletes are currently unknown. The aim of our study was to examine changes in arterial function every 10 km and endothelial function before, 1 h and 24 h after completion of a 50 km ultramarathon.

## 2 METHODS

### 2.1 Ethical approval

All procedures conformed to the latest revision of the *Declaration of Helsinki*, except for registration in a database, and were approved by the Institutional Review Board at the University of Maryland College Park (IRB no. 1300927). All participants submitted written informed consent.

### 2.2 Participants

Eleven experienced adult runners (8 men, 3 women) who signed up to participate in a 50 km trail run were recruited for the current study. Prior to enrolment, interested participants were contacted by phone and asked to confirm that they were over the age of 18 years, had a body mass index (BMI) between 17 and 30 kg/m^2^, were non‐smokers and had no known acute illnesses nor history of CVD. Potential participants were excluded if they could not confirm one or more of the mentioned conditions.

### 2.3 Study design

Participants came to The University of Maryland for two separate visits (visit 1 pre‐race/baseline and visit 2 post‐race) while the race day testing was performed in the field at the race location. Visit 1 (baseline) occurred 7–14 days prior to the day of the race for all the participants, and testing occurred in the morning to align with the initial portion of the race. The race consisted of five 10‐km laps on a course with a total elevation change of ∼762 m over 50 km, an average altitude of ∼120 m and temperatures within the range of 4–8°C. For the race day visit, participants underwent mid‐race measures in a tent located in close proximity to the trail after every 10 km loop, and within 1 h of completing the race. Visit 2 post‐race occurred in the afternoon, 24 h after each participant's race finish time and was performed in the laboratory.

Prior to each laboratory visit, participants were instructed to fast overnight (*≥*10 h). Once in the lab, they were asked to consume the same meal that they planned to eat on race day 30 min before testing began to attempt to control for differences throughout the race due to food intake. This was followed for the pre‐race/baseline and the 24 h post‐race visits. Upon arrival to the laboratory for visit 1, participants gave their written informed consent, completed health history and physical activity questionnaires, were measured for height and weight and underwent air displacement plethysmography using a BodPod® for estimations of body composition (Cosmed USA, Inc., Concord, CA, USA). Next, participants were asked to lie supine for *≥*10 min on a table before flow‐mediated dilatation (FMD) and pulse wave analyses (PWA) were obtained. Following measures of vascular function, participants completed a ${\dot{V}}_{O_{2}}$ maximal exercise test on a treadmill, as described in Landers‐Ramos et al. (2022). Briefly, the maximal exercise test used a constant speed determined by subjects’ preferred running speeds, with 2% increases in incline every 2 min until exhaustion, while ventilation and expired gas concentrations were measured by indirect calorimetry (Cosmed, Rome, Italy). ${\dot{V}}_{O_{2}\max}$ was reached if the O_2_ consumption reached a plateau, and/or if two of the following criteria were met: respiratory exchange ratio >1.10, rating of perceived exertion >18, or a peak heart rate within 10 beats per minute of the participant's age‐predicted maximal heart rate.

### 2.4 Experimental procedures

#### 2.4.1 Flow‐mediated dilatation

Endothelium‐dependent vasodilatation was evaluated via flow‐mediated dilatation (FMD) of the brachial artery at baseline (BL; *n* = 11), 1 h post‐completion of the race (1HP; *n* = 9) and 24 h post‐completion of the race (24HP; *n* = 10). For the BL and 24HP visits, brachial artery diameter was detected with a high‐resolution ultrasound probe (Hitachi‐Aloka Arietta 70, Tokyo, Japan) equipped with a 5–18 MHz linear transducer. At BL and 24HP, image capture was attained with Quipu Cardiovascular Suite FMD Studio (Quipu, Pisa, Italy). The 1HP measurements of FMD were taken at the site of the race using a portable ultrasound instrument (Whale Imaging, Waltham, MA, USA). For all FMD measurements, the ultrasound probe was placed parallel to the left brachial artery and stabilized with a probe holder. To minimize intrasubject variability, the placement of the probe along the participant's arm was kept consistent for each visit. Reactive hyperaemia was induced with the following protocol. A rapid inflator cuff (Hokanson, Bellevue, WA, USA) was placed around the thickest region of the forearm, distal to the antecubital fossa. Baseline arterial diameter and blood velocities were recorded for 60 s. The cuff was then inflated to a suprasystolic pressure (220 mmHg) for 300 s. Arterial diameter and blood velocity were recorded for 150 s post‐cuff deflation to measure endothelial response to reactive hyperaemia. Analyses of all files were conducted using Quipu Cardiovascular Suite FMD Studio. FMD was calculated as the percentage change in arterial diameter from the mean of the 60 s baseline to the peak of the 150 s post‐cuff deflation, as per current FMD guidelines (Limberg et al., 2020). %FMD was allometrically scaled to determine if baseline diameter affected results, as described previously (Mascone et al., 2021). Additionally, FMD was normalized to shear rate (SR) area under the curve (AUC) to peak dilatation.

Reactive hyperaemia (RH) AUC and SR AUC to peak dilatation were assessed via Doppler using pre‐ and post‐cuff occlusion velocity and brachial artery diameter as recorded during the FMD measurement. RH was used to assess microvascular hyperaemic response, calculated using baseline blood flow and post‐occlusion blood flow (150 s following deflation of the forearm cuff):

(1) $$\begin{array}{ccc} \mathrm{Blood}\;\mathrm{flow}\left(\mathrm{mL}/\min\right) & = & \text{Mean blood velocity}\times \pi \\ & & \times \;{\left(\frac{\mathrm{Diameter}\;\left(\mathrm{cm}\right)}{2}\right)}^{2}\times 60 \end{array}$$

SR AUC was used to assess the magnitude of the shear rate stimulus, calculated using the baseline SR and post‐occlusion SR (following deflation of the forearm cuff to peak dilatation):

(2) $$\mathrm{SR}\;\left(s^{-1}\right)=8\times \left(\frac{\mathrm{Mean}\;\mathrm{velocity}\;\left(\mathrm{cm}/s\right)}{\mathrm{Diameter}\;\left(\mathrm{cm}\right)}\right)$$

#### 2.4.2 Pulse wave analysis

Brachial artery pressure waveforms were obtained on the right arm while participants lay supine. These waveforms were produced via propriety software and blood pressure cuffs from SphygmoCor (AtCor Medical, Sydney, Australia). Based on the brachial waveform analyses, brachial systolic (bSBP) and diastolic blood pressures (bDBP) and augmentation index (AIx) were obtained. Similarly, central systolic (cSBP) and diastolic (cDBP) blood pressures were calculated with a generalized transfer function by the software. AIx was adjusted to a heart rate of 75 beats per minute (AIx75) to accommodate for the significant variance in AIx observed among differing heart rates. To determine the aortic forward (*P*_f_) and backward (*P*_b_) pressure waveforms, wave separation analysis was performed with the SphygmoCor's Cardiovascular Suite software. Reflection magnitude (RM) was calculated and reported as the ratio of *P*_b_ to *P*_f_ magnitude and expressed as a percentage [RM = (*P*_b_/*P*_f_) × 100]. The SphygmoCor Xcel system has been reported to produce valid and reliable measurements of AIx/AIx75 and reflection magnitude (Hwang et al., 2014). All pulse wave analysis measurements were obtained at BL, 1HP and 24HP. Additionally, pulse wave analyses were obtained from participants every 10 km throughout the race while subjects lay supine within 5 min of completing each loop.

### 2.5 Statistical analysis

Mixed‐effect ANOVA with factor of time was used to compare the participants’ measures of FMD, shear rate and reactive hyperaemia at BL, 1HP and 24HP and of PWA at BL, 10, 20, 30, 40 km, 1HP and 24HP. Analyses were conducted using Prism (Version 8, GraphPad Software, San Diego, CA, USA). All data are presented as means ± SD unless otherwise specified. Statistical significance was set at *P *< 0.05.

## 3 RESULTS

### 3.1 Participants

Participant characteristics are shown in Table 1. Consistent with many ultramarathon runners (Hoffman & Krishnan, 2013), participants were, on average, middle‐aged and recreationally active as indicated by their ${\dot{V}}_{O_{2}\max}$ (Bergh et al., 1978; Foster et al., 1978; Heath et al., 1981). Participants reported, on average, running continuously over the past 16 ± 12 years, running 5 ± 6 times per week, and running 61.4 ± 30.8 km per week, as reported previously (Landers‐Ramos et al., 2022). Additional race/running history is available in Landers‐Ramos et al. (2022).

**TABLE 1:** Participant characteristics.

Characteristic	Value
Age (years)	40 ± 3
BMI (kg/m^2^)	24.7 ± 1.3
Body fat (%)	18.9 ± 2.2
Resting HR (beats/min)	61.7 ± 2.7
${\dot{V}}_{O_{2}\max}$ (mL/kg/min)	51.4 ± 1.5

*Note*: Values are means ± SD; *n* = 11 participants.

Abbreviations: BMI, body mass index; kg, kilograms; m, meters; HR, heart rate; min, minutes; ${\dot{V}}_{O_{2}\max}$, maximal volume of oxygen consumption; mL; milliliters.

### 3.2 Flow‐mediated dilatation and baseline artery diameter

**FIGURE 1:**
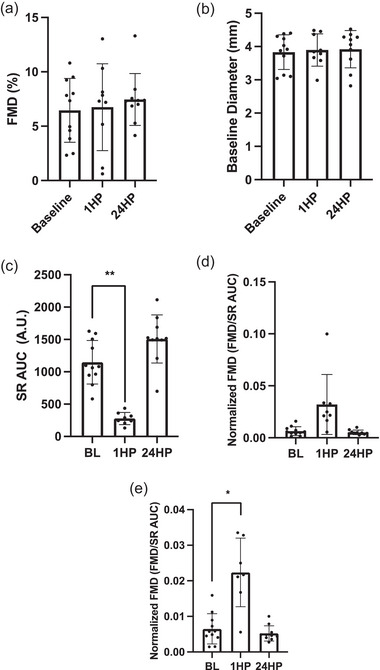
(a) Flow‐mediated dilatation (FMD) at baseline (*n* = 11), 1 h post‐completion (1HP, *n* = 9) and 24 h post‐completion (24HP, *n* = 10). (b) Baseline brachial artery diameter at baseline (*n* = 11), 1HP (*n* = 9) and 24HP (*n* = 10). (c) Peak shear rate area under the curve (Peak SR AUC) at baseline (*n* = 11), 1HP (*n* = 8) and 24HP (*n* = 10). (d) FMD normalized to peak SR AUC with outlier at baseline (*n* = 11), 1HP (*n* = 8) and 24HP (*n* = 10). (e) FMD normalized to peak SR AUC without outlier at baseline (*n* = 11), 1HP (*n* = 7) and 24HP (*n* = 10). Bars indicate means ± SD. Significant difference from baseline: *P < 0.05, **P < 0.001. FMD: 1HP versus BL, *P* = 0.742; 24HP versus BL, *P* = 0.412. Baseline brachial artery diameter: 1HP versus BL, *P* = 0.843; 24HP versus BL, *P* = 0.650. Peak SR AUC: 1HP versus BL, *P *< 0.001; 24HP versus BL, *P* = 0.149. Normalized FMD (with outlier): 1HP versus BL, *P* = 0.067; 24HP versus BL, *P* = 0.697. Normalized FMD (without outlier): 1HP versus BL, *P* = 0.002; 24HP versus BL, *P* = 0.698.

There was no significant difference between FMD or baseline brachial artery diameter at 1HP (*P* = 0.742 and *P* = 0.843, respectively) and 24HP (*P* = 0.412 and *P* = 0.650, respectively) compared with the BL time point (Figure 1). An analysis of FMD normalized to the baseline brachial artery diameter using allometric scaling also revealed no significant differences among time points (data not shown). Images from *n* = 2 participants were not usable at 1HP (*n* = 9 analysed) and *n* = 1 at 24HP (*n* = 10 analysed). Normalized FMD (FMD/SR AUC to peak dilatation) was similar at 1HP and 24HP compared to baseline (*P* = 0.067 and *P* = 0.697, respectively) with an outlier included (*n* = 1 at 1HP). Normalized FMD was significantly higher at 1HP compared to baseline upon removal of the outlier (*P* = 0.002).

### 3.3 Reactive hyperaemia and shear rate to peak dilatation

**FIGURE 2:**
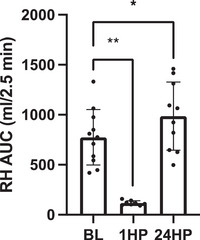
Reactive hyperaemia (RH) area under the curve (AUC) at baseline (BL) (*n* = 11), 1 h post‐completion (1HP) (*n* = 8) and 24 h post‐completion (24HP) (*n *= 10). Bars indicate means ± SD. Significant difference from baseline: *P < 0.05, **P < 0.001. RH AUC: 1HP versus BL, *P* = 0.003; 24HP versus BL, *P* = 0.016. SR AUC: 1HP versus BL, *P *< 0.0001; 24HP versus BL, *P* = 0.055.

Reactive hyperaemia area under the curve (AUC) significantly decreased by 85% at 1HP and significantly increased by 27% at 24HP compared with baseline (*P* = 0.0005 and *P* = 0.016, respectively) (Figure 2). Shear rate AUC significantly decreased by 75% at 1HP (*P* = 0.0006), but not at 24HP (*P* = 0.15) compared with baseline (Figure 1c). Shear rate AUC and reactive hyperaemia AUC were unable to be calculated from *n* = 3 participants at 1HP (*n* = 8 analysed) and *n* = 1 at 24HP (*n* = 10 analysed).

### 3.4 Brachial blood pressure

**FIGURE 3:**
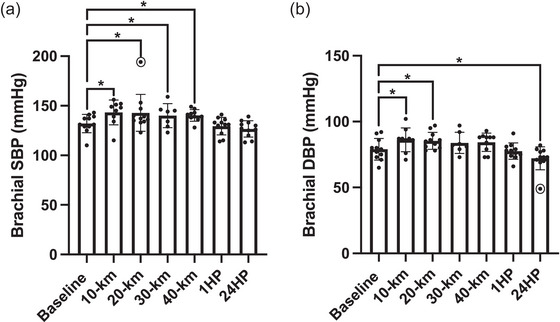
(a) Brachial systolic blood pressure at baseline (*n* = 11), following 10 km (*n* = 9), 20 km (*n* = 11), 30 km (*n* = 7), 40 km (*n* = 10), at 1 h post‐completion (1HP, *n* = 11) and 24 h post‐completion (24HP, *n* = 11). (b) Diastolic blood pressure at baseline (*n* = 11), following 10 km (*n* = 9), 20 km (*n* = 11), 30 km (*n* = 7), 40 km (*n* = 10), at 1HP (*n* = 11) and 24HP (*n* = 11). Bars indicate means ± SD. Significant difference from baseline: *P < 0.05, **P < 0.001. bSBP: 10 km versus BL, *P* = 0.001; 20 km versus BL, *P* = 0.046; 30 km versus BL, *P* = 0.035; 40 km versus BL, *P* = 0.044; 1HP versus BL, *P* = 0.440; 24HP versus BL, *P* = 0.061. bDBP: 10 km versus BL, *P* = 0.007; 20 km versus BL, *P* = 0.023; 30 km versus BL, *P* = 0.188; 40 km versus BL, *P* = 0.138; 1HP versus BL, *P* = 0.576; 24HP versus BL, *P* = 0.004. Note: *n* = 1 outlier for brachial SBP following 20 km and *n *= 1 outlier for brachial DBP at 24 h post‐completion, indicated by circles.

Mean changes in bSBP and bDBP are shown in Figure 3. bSBP significantly increased up to 8% during the race (10 km, *P* = 0.001; 20 km, *P* = 0.046; 30 km, *P* = 0.035; 40 km, *P* = 0.044) compared with BL but was not different from BL after 1HP (*P* = 0.440) nor after 24HP (*P* = 0.061). bDBP significantly increased by 9% following 10 km and increased by 8% following 20 km compared with BL (*P* = 0.007 and *P* = 0.023, respectively). bDBP was similar to BL following 30 km, 40 km and after 1HP (*P* = 0.188, *P* = 0.138 and *P* = 0.576, respectively). After 24HP, the bDBP had significantly decreased by 9% compared with BL (*P* = 0.004). Brachial blood pressure was not obtained from *n *= 2 participants following 10 km (*n* = 9 analysed), *n* = 4 participants following 30 km (*n* = 7 analysed), and *n* = 1 following 40 km (*n* = 10 analysed).

### 3.5 Central blood pressure

**FIGURE 4:**
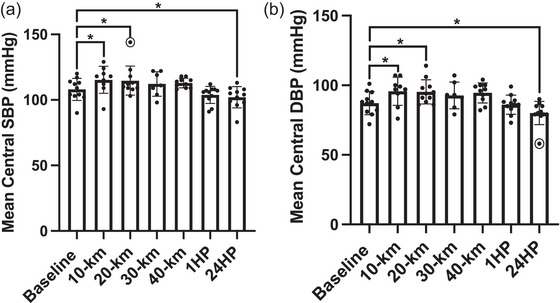
(a) Systolic blood pressure at baseline (*n* = 11), following 10 km (*n* = 9), 20 km (*n* = 11), 30 km (*n* = 7), 40 km (*n* = 10), at 1 h post‐completion (1HP, *n* = 11) and 24 h post‐completion (24HP, *n* = 11). (b) Central diastolic blood pressure at baseline (*n* = 11), following 10 km (*n* = 9), 20 km (*n* = 11), 30 km (*n* = 7), 40 km (*n* = 10), at 1HP (*n* = 11) and 24HP (*n* = 11). Bars indicate means ± SD. Significant difference from baseline: *P < 0.05, **P < 0.001. cSBP: 10 km versus BL, *P* = 0.004; 20 km versus BL, *P* = 0.034; 30 km versus BL, *P* = 0.145; 40 km versus BL, *P* = 0.121; 1HP versus BL, *P* = 0.104; 24HP versus BL, *P* = 0.006. bDBP: 10 km versus BL, *P* = 0.003; 20 km versus BL, *P* = 0.009; 30 km versus BL, *P* = 0.182; 40 km versus BL, *P* = 0.061; 1HP versus BL, *P* = 0.695; 24HP versus BL, *P* = 0.002. Note: *n* = 1 outlier for mean central SBP following 20 km and *n* = 1 outlier for mean central DBP at 24 host completion, indicated by circles.

Mean changes in cSBP and cDBP are shown in Figure 4. There was a significant increase in cSBP by 7% following 10 km and 6% following 20 km compared to BL (*P* = 0.004 and *P* = 0.034, respectively). cSBP was similar to BL following 30 km, 40 km and at 1HP (*P* = 0.145, *P* = 0.121 and *P* = 0.104, respectively). cSBP significantly decreased by 5% at 24HP compared to BL (*P* = 0.006). cDBP increased by 10% following 10 km and by 9% following 20 km compared to BL (*P* = 0.003 and *P* = 0.009, respectively). cDBP was similar to BL following 30 km, 40 km and at 1HP (*P* = 0.182, *P* = 0.061 and *P* = 0.695, respectively). cDBP significantly decreased by 8% at 24HP compared to BL (*P* = 0.002). Brachial blood pressure was not obtained from *n* = 2 participants following 10 km (*n* = 9 analysed), *n* = 4 participants following 30 km (*n* = 7 analysed) and *n* = 1 following 40 km (*n* = 10 analysed).

### 3.6 Augmentation index

**FIGURE 5:**
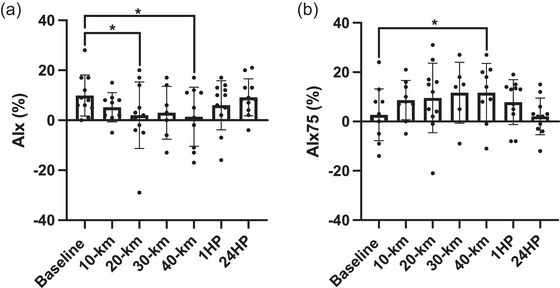
(a) Augmentation index at baseline (*n* = 11), following 10 km (*n* = 9), 20 km (*n* = 11), 30 km (*n* = 7), 40 km (*n* = 10), at 1 h post‐completion (1HP, *n* = 11) and 24 h post‐completion (24HP, *n* = 11). (b) Augmentation index standardized to a heart rate of 75 beats/min at baseline (*n* = 11), following 10 km (*n* = 9), 20 km (*n* = 11), 30 km (*n* = 6), 40 km (*n* = 9), at 1HP (*n* = 11) and 24HP (*n* = 11). Bars indicate means ± SD. Significant difference from baseline: *P < 0.05, **P < 0.001. AIx: 10 km versus BL, *P* = 0.114; 20 km versus BL, *P* = 0.027; 30 km versus BL, *P* = 0.069; 40 km versus BL, *P* = 0.006; 1HP versus BL, *P* = 0.196; 24HP versus BL, *P* = 0.654. AIx75: 10 km versus BL, *P* = 0.060; 20 km versus BL, *P* = 0.050; 30 km versus BL, *P* = 0.056; 40 km versus BL, *P* = 0.020; 1HP versus BL, *P* = 0.089; 24HP versus BL, *P* = 0.739.

Mean changes in AIx and AIx75 are shown in Figure 5. AIx remained similar following 10 km (*P* = 0.114), significantly decreased following 20 km (*P* = 0.027), was similar following 30 km and significantly decreased following 40 km (*P* = 0.006) compared to BL. AIx was similar to BL after 1HP and 24HP (*P* = 0.196 and *P* = 0.654, respectively). AIx75 was similar following 10, 20 and 30 km, significantly increasing following 40 km compared with BL (*P* = 0.060, *P* = 0.050, *P* = 0.056 and *P* = 0.020, respectively). AIx75 was similar after 1HP and 24HP compared to BL (*P* = 0.089 and *P* = 0.739, respectively). AIx was not obtained from *n* = 2 participants following 10 km (*n* = 9 analysed), *n* = 4 participants following 30 km (*n* = 7 analysed) and *n* = 1 participants following 40 km (*n* = 10 analysed). AIx75 was not obtained from *n* = 2 participants following 10 km (*n* = 9 analysed), *n* = 5 participants following 30 km (*n* = 6 analysed) and *n* = 2 participants following 40 km (*n* = 9 analysed).

### 3.7 Wave reflection

**FIGURE 6:**
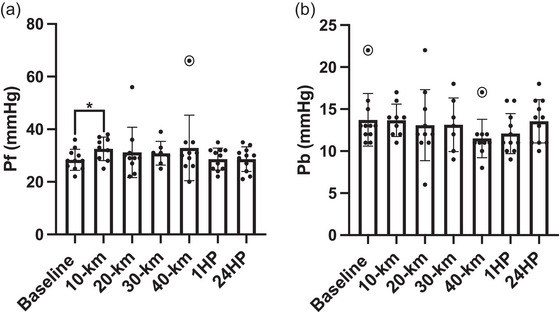
(a) Forward wave amplitude at baseline (*n* = 11), following 10 km (*n* = 9), 20 km (*n* = 10), 30 km (*n* = 7), 40 km (*n* = 10), at 1 h post‐completion (1HP, *n* = 11) and 24 h post‐completion (24HP, *n* = 11). (b) Backward wave reflection at baseline (*n* = 11), following 10 km (*n* = 9), 20 km (*n* = 10), 30 km (*n* = 7), 40 km (*n* = 10), at 1HP (*n* = 11) and 24HP (*n* = 11). Bars indicate means ± SD. Significant difference from baseline: *P < 0.05, **P < 0.001. Note: *n* = 1 outlier for forward wave amplitude following 40 km and *n* = 1 outlier for backward wave reflection at baseline and *n* = 1 outlier following 40 km, as indicated by circles. *P*_f_: 10 km versus BL, *P* = 0.049; 20 km versus BL, *P* = 0.389; 30 km versus BL, *P* = 0.429; 40 km versus BL, *P* = 0.358; 1HP versus BL, *P* = 0.908; 24HP versus BL, *P* = 0.897. *P*_b_: 10 km versus BL, *P* = 0.957; 20 km versus BL, *P* = 0.706; 30 km versus BL, *P* = 0.753; 40 km versus BL, *P* = 0.084; 1HP versus BL, *P* = 0.223; 24HP versus BL, *P* = 0.849.

Changes in the variables of wave reflection are shown in Figure 6. Forward wave amplitude (*P*_f_) significantly increased by 15% following 10 km (*P* = 0.049) but was similar to baseline following 20, 30, 40 km, 1HP and 24HP (*P* = 0.389, *P* = 0.429, *P* = 0.358, *P* = 0.908 and *P* = 0.897, respectively). Backward wave reflection (*P*_b_) did not significantly change from BL, remaining similar following 10, 20, 30, 40 km, 1HP and 24HP (*P* = 0.957, *P* = 0.706, *P* = 0.753, *P* = 0.084, *P* = 0.223 and *P* = 0.849, respectively). Reflected magnitude (RM %) did not significantly change from BL, remaining similar following 10, 20, 30, 40 km, 1HP and 24HP (*P* = 0.139, *P* = 0.124, *P* = 0.513, *P* = 0.237, *P* = 0.354 and *P* = 0.997, respectively). Wave reflection was not obtained from *n* = 2 participants following 10 km (*n *= 9 analysed), *n* = 1 participant following 20 km (*n *= 10 analysed), *n* = 4 participants following 30 km (*n* = 7 analysed) and *n* = 1 participants following 40 km (*n* = 10 analysed).

### 3.8 Heart rate

**FIGURE 7:**
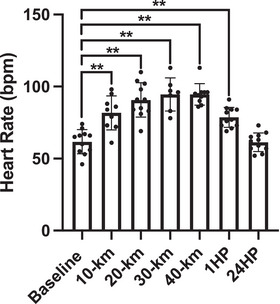
Heart rate at baseline (*n* = 11), following 10 km (*n* = 9), 20 km (*n* = 11), 30 km (*n* = 7), 40 km (*n* = 10), at 1 h post‐completion (1HP, *n* = 11) and 24 h post‐completion (24HP, *n* = 11). Bars indicate means ± SD. Significant difference from baseline: *P < 0.05, **P < 0.001. HR: 10 km versus BL, *P* = 0.0006; 20 km versus BL, *P *< 0.0001; 30 km versus BL, *P *< 0.0001; 40 km versus BL, *P *< 0.0001; 1HP versus BL, *P* = 0.0008; 24HP versus BL, *P* = 0.866.

Mean changes in heart rate are shown in Figure 7. Heart rate significantly increased throughout the race, reaching up to 53% higher following 30 and 40 km compared with BL (10 km, *P* = 0.0006; 20 km, *P *< 0.0001; 30 km, *P *< 0.0001; 40 km, *P *< 0.0001). HR remained 27% higher following 1HP compared with BL (*P* = 0.0008), but remained similar to BL at 24HP (*P* = 0.866). Heart rate was not obtained for *n* = 2 participants following 10 km (*n* = 9 analysed), *n* = 4 following 30 km (*n* = 7 analysed) and *n *= 1 following 40 km (*n* = 10 analysed).

## 4 DISCUSSION

The novel findings in the present study were (1) there were no significant changes in %FMD 1 and 24 h after a 50 km ultramarathon compared with baseline, even though there was a decrease in microvascular function immediately following and 24 h after the race; (2) forward wave magnitude significantly increased following 10 km, but returned to baseline following 20 km whereas backward wave magnitude and reflection magnitude remained similar to baseline; and (3) AIx75 significantly increased following 40 km, but was similar to baseline by 1 h after the race. We have previously reported an increase in inflammation following this 50 km race as shown in Table 2 (Landers‐Ramos et al., 2022) in agreement with previous studies on prolonged running (Kasprowicz et al., 2013; Nieman et al., 2000). Although systemic inflammation was present (Gill et al., 2015; Marklund et al., 2013), there were no significant decreases in any macrovascular function measure post‐race compared with baseline.

**TABLE 2:** Plasma cytokines in response to a 50 km race.

	Baseline	10 km	1 HP	24 HP
IL‐6 (pg/mL)	0.28 ± 0.24	1.1 ± 0.81^a^	7.5 ± 2.6^a,b^	0.52 ± 0.23^a,b,c^
IL‐8 (pg/mL)	4.3 ± 3.2	6.8 ± 6.2^a^	11.1 ± 11.6^a^	3.9 ± 3.5^b,c^
Calprotectin (ng/mL)	809.8 ± 316.2	1214.3 ± 120.8^a^	1362.5 ± 63.9^a,b^	804.7 ± 250.4^b,c^
TNF‐α (pg/mL)	0.77 ± 0.25	0.79 ± 0.33	0.82 ± 0.24	0.74 ± 0.18

*Note*: Values are means ± SD. Results from IL‐6, calprotectin and TNF‐α were reported in Landers‐Ramos et al. (2022) and are presented here to provide context to the vascular outcomes. ^a^Significant difference from BL. ^b^Significant difference from 10 km. ^c^Significant difference from 1HP. Abbreviations: IL, interleukin; pg, picogram; mL, milliliter; ng, nanogram; km, kilometers; HP, hours post‐race; TNF, tumour necrosis factor.

In the current study, the preservation of macrovascular function could be explained by variation in NO‐mediated vasodilatation occurring in the active versus inactive muscular beds (Green et al., 2012). It is possible that a primarily lower‐limb exercise, such as prolonged running, produces differential flow and/or shear rate patterns when compared with those of a primarily upper‐limb exercise. This is supported by the findings from Dawson et al. (2008) in which marathon running (42.2 km) reduced femoral %FMD, but not brachial %FMD immediately following the race. Along the lines of shear rate, the shear rate AUC to peak dilatation was significantly lower 1HP (Figure 1c), and therefore when FMD was normalized to shear rate AUC (to peak dilatation without outlier), it was significantly greater at 1HP (Figure 1e). Active muscle is also known to release local metabolites that promote vasodilatation of small blood vessels within the muscle (Halliwill et al., 2013). Thus, in addition to potential limb‐specific responses, changes in microvascular function may be more apparent in response to this type of exercise. Surprisingly, the %FMD also did not appear to be affected by the systemic inflammation at 1HP, reflected by the significant increase in IL‐6 compared with baseline (Landers‐Ramos et al., 2022). Furthermore, %FMD was also not affected by the elevated IL‐6 levels at 24HP either.

An important variable that could affect FMD is the baseline diameter of the artery. Along these lines, in the current study, the FMD baseline diameter also did not significantly change 1 and 24 h post‐race compared with the FMD baseline diameter at the baseline time point. It is plausible that given the training status of the participants and the regularity of these races for some of the participants, their arterial diameter may already be structurally larger to accommodate for the repetitive functional responsiveness needed to maintain blood pressure during exercise, as shown in previous research on endurance runners (Black et al., 2016); therefore, the percentage change in the diameter would not be to the same degree as an untrained individual (Green et al., 2012). Furthermore, as RH is variable between individuals or following a stressor, the stimulus–response relationship in FMD needs to be interpreted critically. In this context, a minor change in FMD could be related to the resistance artery function as opposed to a macrovascular dysfunction (Ku et al., 2014; Mitchell et al., 2004; Wray et al., 2006).

Reactive hyperaemia and resulting shear stress (rate) are the stimuli for vasodilatation during FMD. The reactive hyperaemic response has recently been suggested to reflect microvascular function (Rosenberry & Nelson, 2020). The structural and functional adaptations that accompany endurance training could also explain the lack of changes in %FMD even though the reactive hyperaemia AUC decreased 1HP, potentially indicating a transient decrease in microvascular function. One of the potential explanations for the significant decrease in microvascular function could be vasoconstriction within the upper‐body peripheral arteries, due to sympathetic modulation. Furthermore, it is plausible that the increases in the acute inflammation during and at 1HP affected the microcirculation at a greater degree compared with the conduit artery. The current study suggests the microcirculation may be more responsive and sensitive to the systemic changes compared with the conduit artery in response to exercise. This is in contrast to a previous study by King et al. (2021) in which RH AUC remained similar following a 50 km race; however, RH AUC was measured in the femoral artery, inflammation remained similar following the 50 km race and the post‐time measurement varied, which could account for these differences (King et al., 2021).

Previously, Burr et al. (2014) reported a decrease in large artery compliance in ultramarathoners compared with age‐matched controls. Interestingly, the data from the current study suggest a significant decrease in the wave reflection as measured by the change in AIx from baseline following 20 and 40 km. Taken together, a reduction in wave‐reflection compared with baseline could be likely due to the matching of the impedance between the large and the peripheral vasculature (Vyas et al., 2007). As opposed to regular healthy individuals and data from Burr et al. (2014), it is plausible that the ultramarathon runners in the current study had a more compliant aorta compared with the peripheral vasculature and thus have a greater mismatch at rest. In response to the increasing HR and increased pulse wave velocity while running, the impedance within the aorta and that of the peripheral vasculature matches causing a decrease in wave reflection. Although significant caution is warranted in interpretation of AIx as a pure measure of wave‐reflection and impedance especially given that hormonal, autonomic and arterial structure could significantly affect impedance within a closed‐loop system. Along these lines, the data from the change values for AIx75 (wave reflection normalized to fixed heart rate) between baseline and after 40 km were significantly increased compared with change values between baseline and 1 h post‐race. Arterial compliance has been shown to decrease after prolonged endurance bouts, including ultramarathons (Bonsignore et al., 2017; Burr et al., 2012). Inherent association between increased arterial stiffness and amplified wave reflection may, therefore, explain the rise in AIx75 observed following 40 km in the present study.

A potential limitation of the current study was the failure to measure pulse wave velocity, which is the gold standard for measuring arterial stiffness. It was not logistically possible to measure pulse wave velocity during the ultramarathon without significantly affecting the race times for the runners. Additionally, it should be noted that all participants included in the present investigation were experienced/habitual runners. We speculate that sedentary individuals, or those habitually exercising at lower durations and intensities than the cohort of endurance‐trained recreational runners, may have different responses of cardiovascular structure and function throughout the 24 h recovery window from those seen in our study. In addition, the race length and duration of this study are shorter than in previous ultramarathon studies (Bonsignore et al., 2017; Burr et al., 2012). Post‐race arterial stiffness has also differed in previous studies based on the length of the race. For example, in a study by Bonsignore et al. (2017) there was an increase in arterial compliance after an 80 km ultramarathon, but a decrease in arterial compliance after a 195 km ultramarathon. It is possible there is a dose–response relationship between the duration of the race and arterial compliance. Therefore, the shorter duration of this race (50 km) could affect the comparison to previous ultramarathon studies, though this distance is more applicable to most ultramarathon runners.

### 4.1 Conclusions

In the current study we demonstrated that macrovascular function as measured by FMD in the brachial artery remains unchanged after a 50 km ultramarathon. Interestingly, even though the macrovascular function remained unchanged, the 50 km ultramarathon did increase inflammation and lower the microvascular function. However, the microvascular changes lasted less than 24 h post‐race suggesting the ultramarathon distance of 50 km has relatively short‐term effects in trained individuals.

## AUTHOR CONTRIBUTIONS

All authors contributed equally to this manuscript: Cynthia M. Weiner—conception of the manuscript, interpretation of results and drafting and revising; Sushant M. Ranadive—conception and design, acquisition and interpretation, revising; Lauren E. Eagan—design, acquisition and analysis, revising; Odessa Addison—conception and design, acquisition and interpretation, revising; Rian Q. Landers‐Ramos—conception and design, acquisition and interpretation, revising; Steven J. Prior—conception and design, acquisition and interpretation, revising. All authors approved the final version of the manuscript and agree to be accountable for all aspects of the work in ensuring that questions related to the accuracy or integrity of any part of the work are appropriately investigated and resolved. All persons designated as authors qualify for authorship, and all those who qualify for authorship are listed.

## CONFLICT OF INTEREST

The authors declare that there are no conflicts of interest to disclose.

## Data Availability

The data that support the findings of this study are available from the corresponding author, S.M.R., upon reasonable request.
